# PLA2G16 Expression in Human Osteosarcoma Is Associated with Pulmonary Metastasis and Poor Prognosis

**DOI:** 10.1371/journal.pone.0127236

**Published:** 2015-05-18

**Authors:** Shoulei Liang, Zhiwu Ren, Xiuxin Han, Jilong Yang, Luling Shan, Lin Li, Binying Wang, Qianyi Zhang, Tianyang Mu, Kexin Chen, Shunbin Xiong, Guowen Wang

**Affiliations:** 1 Department of Bone and Soft Tissue Tumors, Tianjin Medical University Cancer Institute and Hospital, National Clinical Research Center for Cancer, Key Laboratory of Cancer Prevention and Therapy, Tianjin, China; 2 Institute of Cancer Stem Cell, Dalian Medical University Cancer Center, Dalian, China; 3 School of Ophthalmology & Optometry, Wenzhou Medical University, Wenzhou, China; 4 Department of Epidemiology and Biostatistics, Tianjin Medical University Cancer Institute and Hospital, Tianjin, China; 5 Department of Genetics, The University of Texas, M.D. Anderson Cancer Center, Houston, Texas, United States of America; University of North Carolina School of Medicine, UNITED STATES

## Abstract

**Background:**

Osteosarcoma is the most frequent type of malignant bone tumor in children and adolescents and is associated with a high propensity for lung metastasis. Recent experiments have indicated that PLA2G16 contributes to osteosarcoma progression and metastasis in both mouse and human osteosarcoma cell lines. The aim of this study was to compare the expression of PLA2G16 in non-metastatic and metastatic osteosarcomas to determine whether PLA2G16 expression can serve as a biomarker of osteosarcoma prognosis and metastasis.

**Methods:**

Quantitative real-time PCR was used to examine *PLA2G16* mRNA in primary osteosarcoma patients (18 patients without metastases and 17 patients with metastases), and immunohistochemistry (IHC) staining of PLA2G16 was performed on tissue microarrays from 119 osteosarcoma patients. Tumor metastatic behavior and survival of the patients were followed up for a minimum of 36 months and a maximum of 171 months. The prognostic value of PLA2G16 expression was evaluated by the Kaplan–Meier method and a log-rank test. Multivariate Cox regression analysis was used to identify significant independent prognostic factors.

**Results:**

Osteosarcoma patients with metastasis showed a higher expression of PLA2G16 at both the mRNA and protein levels (both at P values< 0.05) than did patients without metastasis. Osteosarcoma patients with positive IHC staining of PLA2G16 expression at primary sites had shorter overall survival and metastasis-free survival (both at P values <0.02). Moreover, multivariate Cox analysis identified PLA2G16 expression as an independent prognostic factor to predict poor overall survival and metastasis-free survival (both P values < 0.03).

**Conclusions:**

This study indicated that PLA2G16 expression is a significant prognostic factor in primary osteosarcoma patients for predicting the development of metastases and poor survival.

## Introduction

Worldwide, osteosarcoma is the most frequent primary solid malignant bone tumor in adolescents and young adults [1]. It usually involves long bones and is a highly aggressive tumor that metastasizes primarily to the lungs. The prognosis for patients with metastatic osteosarcoma remains poor with a 5-year survival rate at only 10 to 20%, despite aggressive multi-modality therapy [2, 3]. Thus, it is highly desirable to identify novel targets and develop new strategies that inhibit lung metastasis from the primary osteosarcoma site.

Recently, PLA2G16 has been shown to contribute to osteosarcoma progression and metastasis in both mouse and human osteosarcoma cell lines [4]. PLA2G16 is classified as a Group XVI phospholipase A_2_ (PLA2G16) and is expressed in most normal tissues [5, 6]. The enzymatic activity of PLA2G16 hydrolyzes the ester bond at the sn-2 position of membrane phospholipids, preferably phosphatidylcholine, and releases free fatty acids (FFA) and lysophospholipid [5], both of which increase proliferation, migration, and metastasis[7–10]. Previously, PLA2G16 was identified as a class II tumor suppressor because it inhibited *H-ras*-induced transformation [11] and its expression was lost in some human tumor types, including breast, ovary, kidney and testicular germ cell [6, 12]. However, a recent study indicated that PLA2G16 may act to promote cellular growth in a subset of non-small cell lung carcinomas [13]. Furthermore, Cancer Profiling Array I analysis showed that the PLA2G16 expression levels were increased in not only lung but also colon, stomach, and rectum cancers, suggesting that PLA2G16 can act as an oncogene in these tumors [13]. These findings suggested that PLA2G16 may play different roles in various types of malignancies. In this study, we examined PLA2G16 expression in human osteosarcoma patients by quantitative real-time PCR (qRT-PCR) and immunohistochemistry (IHC) assays and determined whether the expression of PLA2G16 can be used as a prognostic or metastasis marker of human osteosarcoma.

## Material and Methods

### Ethics statement

The research ethics committee of the Cancer Institute and Hospital of Tianjin Medical University (China) provided ethical approval for this study, and all patients provided written informed consent. All specimens were handled and stored anonymously according to ethical and legal standards.

### Patients and Samples

Fresh osteosarcoma tissue samples (n = 35) were obtained from 18 primary osteosarcoma patients without metastases and 17 patients with metastases. All patients presented at the Tianjin Medical University Cancer Institute and Hospital between 2006 and 2014.Diagnoses were confirmed by pathological examination. None of the patients had received radiotherapy or chemotherapy before surgery. These tissue samples were frozen in liquid nitrogen and kept at -80°C until RNA extraction.

Primary tumor samples were collected from 119 patients who had undergone surgical treatment for osteosarcoma with pathologic identification in the Cancer Institute and Hospital of Tianjin Medical University (China) from 1996 to 2011.Patients were enrolled in this retrospective study based on the following criteria: diagnosis of osteosarcoma with histopathological assessment, no prior anticancer treatment, and the availability of complete clinicopathologic and follow-up data. Tumor tissue specimens were grouped according to the sixth edition of the TNM classification of the International Union against Cancer (UICC).

### Quantitative real-time PCR

The total RNA was extracted and purified with TRIzol reagent (Life Technologies, NY, USA) according to the manufacturer’s instructions. A parallel tube without RT (RT-negative control) was included in the RT reactions and subsequent TaqMan PCR procedures as the control for possible DNA contamination.

The reverse transcription of RNA to cDNA was conducted using a High Capacity cDNA Archive Kit (Applied Biosystems, Foster City, CA). One hundred nanograms of total cDNA were added per 20 μl reaction with sequence-specific primers and Taqman probes. Quantitative gene expression was analyzed for PLA2G16 (Hs00912734_m1) and GAPDH (Hs02758991_g1) with gene-specific probes (Applied Biosystems) using Taqman Universal PCR Master Mix and was carried out in triplicate on an ABI Prism 7900 system according to the manufacturer’s instructions. The data were then quantified using the comparative Ct method for relative gene expression compared with GAPDH as endogenous control.

### Tissue Microarrays and Immunohistochemistry

Tissue Microarrays (TMAs) were constructed as previously described [14]. Briefly, samples were fixed in 4% buffered formalin, decalcified using EDTA if required, and embedded in paraffin to construct TMAs. Thick portions of tumors were serially sectioned (4 mm), with the most representative areas of the tumor region carefully selected and sampled for the TMA collector blocks. To validate the concordance between TMAs and whole tumor sections, we further detected PLA2G16 expression for 20 patients randomly chosen from the 119 patients for comparison with whole tumor sections.

Immunohistochemical examination of PLA2G16 was performed with polyclonal PLA2G16 antibodies (No.10337, Cayman chemical) at a 5ug/ml dilution. Antigen retrieval was performed by high-voltage plerosis in Tris (0.01 mmol/L, pH = 6.0) and then EDTA buffer solution (1 mmol/L, pH = 9.0) for 150 s. After deparaffinization, rehydration, and heat-induced antigen retrieval, tissues were incubated with primary antibodies overnight at 4°C.After incubation for 30 minutes with the secondary antibody, the sections were developed in diaminobenzidine solution under microscopic observation and counterstained with hematoxylin. Negative control slides with the primary antibodies omitted were included for all assays.

IHC staining was evaluated by two independent pathologists who were blinded to the clinicopathological parameters and clinical outcomes of the patients. The staining was determined semi-quantitatively according to the intensity (0 = no staining, 1 = weak staining, 2 = moderate staining, 3 = strong staining) and the percentage of positive cells (0: none or <5%; 1: 5% to 20%; 2: 21% to 40%; 3: >40%). Scores of 0 to 2 were considered negative, and scores of 3 to 6 were considered positive. Cells were counted in at least three fields (at ×400 magnification) in the tumor areas.

### Statistical Analysis

The differences in the*PLA2G16* mRNA expression levels between primary non-metastatic osteosarcoma samples and the metastatic osteosarcoma samples were analyzed using Student’s t-test. For the IHC analysis, the correlation of PLA2G16 expression with the clinicopathologic data was analyzed using a Chi-square test. Patient survival curves were plotted according to the Kaplan—Meier method and a log-rank test. Multivariate Cox regression analysis was used to identify significant independent prognostic factors. The overall survival was defined as the time period from the date of diagnosis to that of death or the last follow-up. For the metastasis-free survival (MFS) analysis, the duration was defined as the time from diagnosis until the occurrence of metastasis. If these patients had metastatic disease at diagnosis, the event was considered time 0.A two-sided P value <0.05 was considered statistically significant. All statistical analyses were carried out using the SPSS version 18.0 statistical software (SPSS, Chicago, IL).

## Results

### Expression of *PLA2G16* mRNA in human non-metastatic and metastatic primary osteosarcoma samples

**Fig 1:**
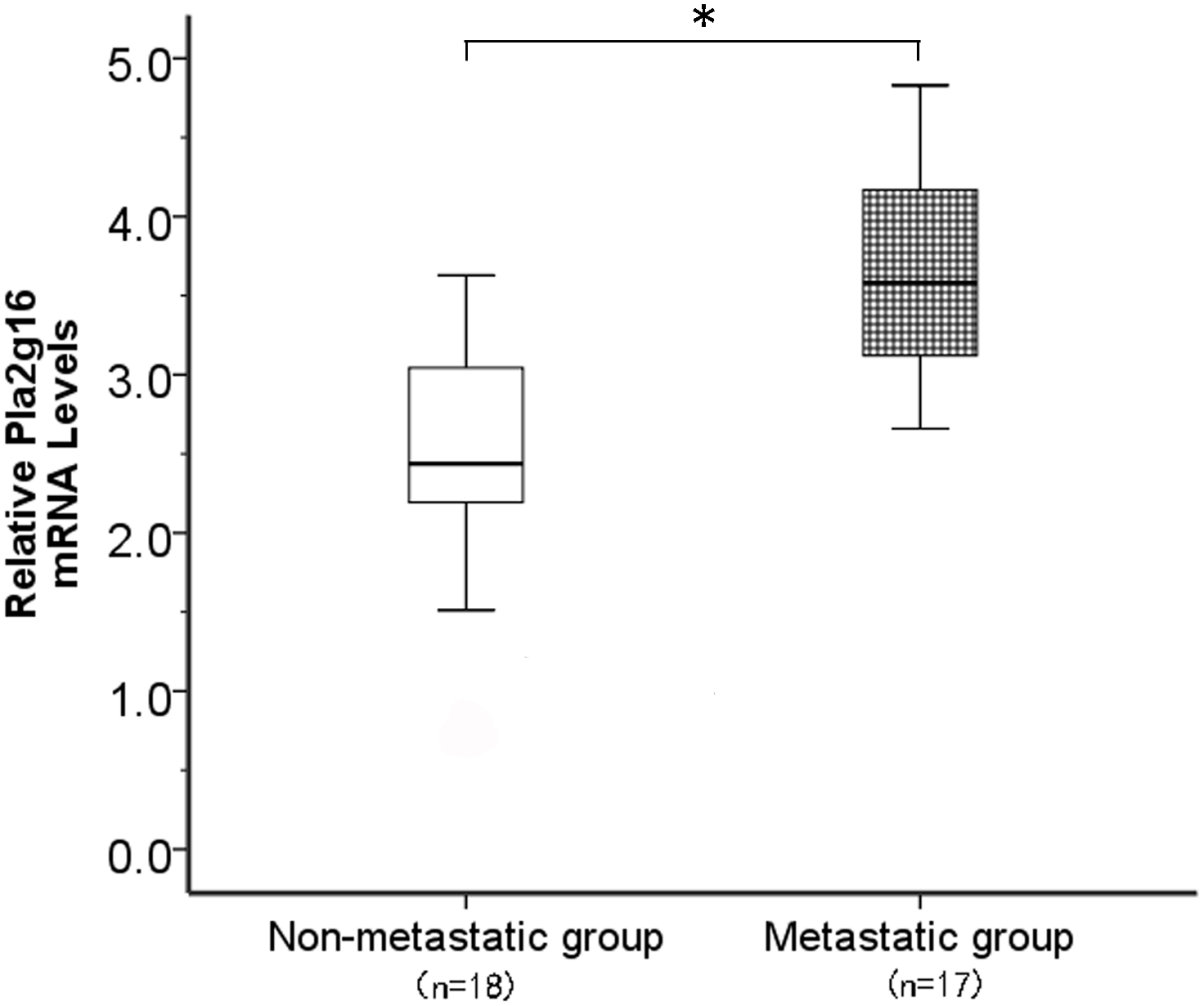
PLA2G16 expression in osteosarcoma tumors with or without metastasis The PLA2G16 mRNA levels were determined by real-time quantitative PCR. *denote p values P<0.05.

To examine the expression of *PLA2G16* mRNA in fresh osteosarcoma patient samples, we harvested 18 primary tumor samples without metastasis and 17 primary tumor samples with metastasis. According to the qRT-PCR analysis, the expression levels of *PLA2G16* mRNA were found to be significantly increased by 1.47-fold, on average, in the metastatic group compared with the non-metastatic group (Fig 1). The statistical analysis showed that the relative level of *PLA2G16* mRNA expression in metastatic primary osteosarcoma tissues (mean ± SD: 3.65 ± 0.60) was clearly higher than that in non-metastatic tissues (mean ± SD: 2.49± 0.69; P < 0.05, Fig 1). These data suggested that the higher expression level of *PLA2G16* in osteosarcoma was associated with metastasis.

### Association of IHC staining for PLA2G16 with clinicopathologic variables in osteosarcoma patients

To examine the expression of PLA2G16 in human osteosarcoma patients at the protein level, we collected 119 osteosarcoma patient samples and made a TMA of the samples. Seventy-three male and 46 female patients were included in the study. Follow-up was available for all patients, with a median time of 94 months (range: 2 months to 171 months).Sixty patients (60/119, 50.4%) died during the follow-up period, primarily due to metastases (45/60, 75%). Fifty patients (50/119, 42%) developed metastases at a mean of 14.5 months (range 0–65 months). Of these patients, 41 had metastases in the lung, and four had metastases in bones (five patients had both lung and bone metastases).The median overall and metastasis-free survival times were 86 months (95% confidence interval [CI], 37.5–136.5 months) and 34.9 months (95% CI, 17.6–154.4 months), respectively. The clinical and histopathological details of the 119 cases are listed in Tables 1 and 2 and S1 Table.

**Table 1:** Relationship between PLA2G16 and Clinicopathologic Factors of Patients.

Variables	PLA2G16 expression (N)				
	Total (N = 119)	Positive	Negative	X^2^	P-values
Gender					
Male	73(61.3%)	41	32	0.181	0.670
Female	46(38.7%)	24	22		
Age					
≤ 20 years	68(57.1%)	36	32	0.181	0.671
>20 years	51(42.9%)	29	22		
Tumor Location					
Femur	56(47.1%)	25	31	8.435	0.077
Tibia	24(20.2%)	13	11		
Humerus	13(10.9%)	11	2		
Fibula	11(9.2%)	8	3		
Others	15(12.6%)	8	7		
Histological classification					
Osteoblastic	79(66.4%)	44	35	0.966	0.617
Chondroblastic	22(18.5%)	13	9		
Others	18(15.1%)	8	10		
Metastasis					
Yes	50(42.0%)	38	12	15.900	P<0.001
No	69(58.0%)	27	42		
HIstological grade					
I	34(28.6%)	16	18	1.586	0.452
II	53(44.5%)	29	24		
III	32(26.9%)	20	12		
Enneking staging					
I	12(10.1%)	4	8	4.812	0.186
II A	25(21.0%)	16	9		
II B	71(69.7%)	37	34		
III	11(9.2%)	8	3		

Abbreviation: PLA2G16, Group XVI phospholipase A_2_.

**Table 2:** Clinicopathologic patient characteristics and univariate survival analysis.

Variable	Patients (n = 119)	3-y OS Rate (%)	P value	3-y MFS Rate (%)	P value
Male	73	64.4%	0.118	52.8%	0.329
Female	46	82.6%		62.3%	
Age at diagnosis					
≤ 20 years	68	75.0%	0.613	62.6%	0.634
> 20 years	51	66.67%		60.0%	
Tumor location					
Femur	56	75.0%	0.608	63.2%	0.693
Tibia	24	66.7%		49.8%	
Humerus	13	61.5%		44.9%	
Fibula	11	81.8%		63.6%	
Others	15	66.7%		53.3%	
Histological classification					
Osteoblastic	79	69.6%	0.459	56.1%	0.570
Chondroblastic	22	63.6%		54.2%	
Others	18	88.9%		66.2%	
Lung metastasis					
Yes	50	42.0%	<0.001	17.6	<0.001
No	69	92.8%		82.6	
Histological grade					
I	34	88.2%	0.005	81.7%	0.005
II	53	66.0%		57.9%	
III	32	52.9%		40.0%	
Enneking staging					
I	12	100%	<0.001	100%	<0.001
II A	25	76.0%		63.3%	
II B	71	68.9%		59.9%	
III	11	18.2%		0	
PLA2G16					
Negative	54	75.7%	0.013	74.0%	0.008
Positive	65	63.1%		47.4%	

Abbreviation: OS, Overall survival; MFS, Metastasis-free survival; PLA2G16, Group XVI phospholipase A_2_.

**Fig 2:**
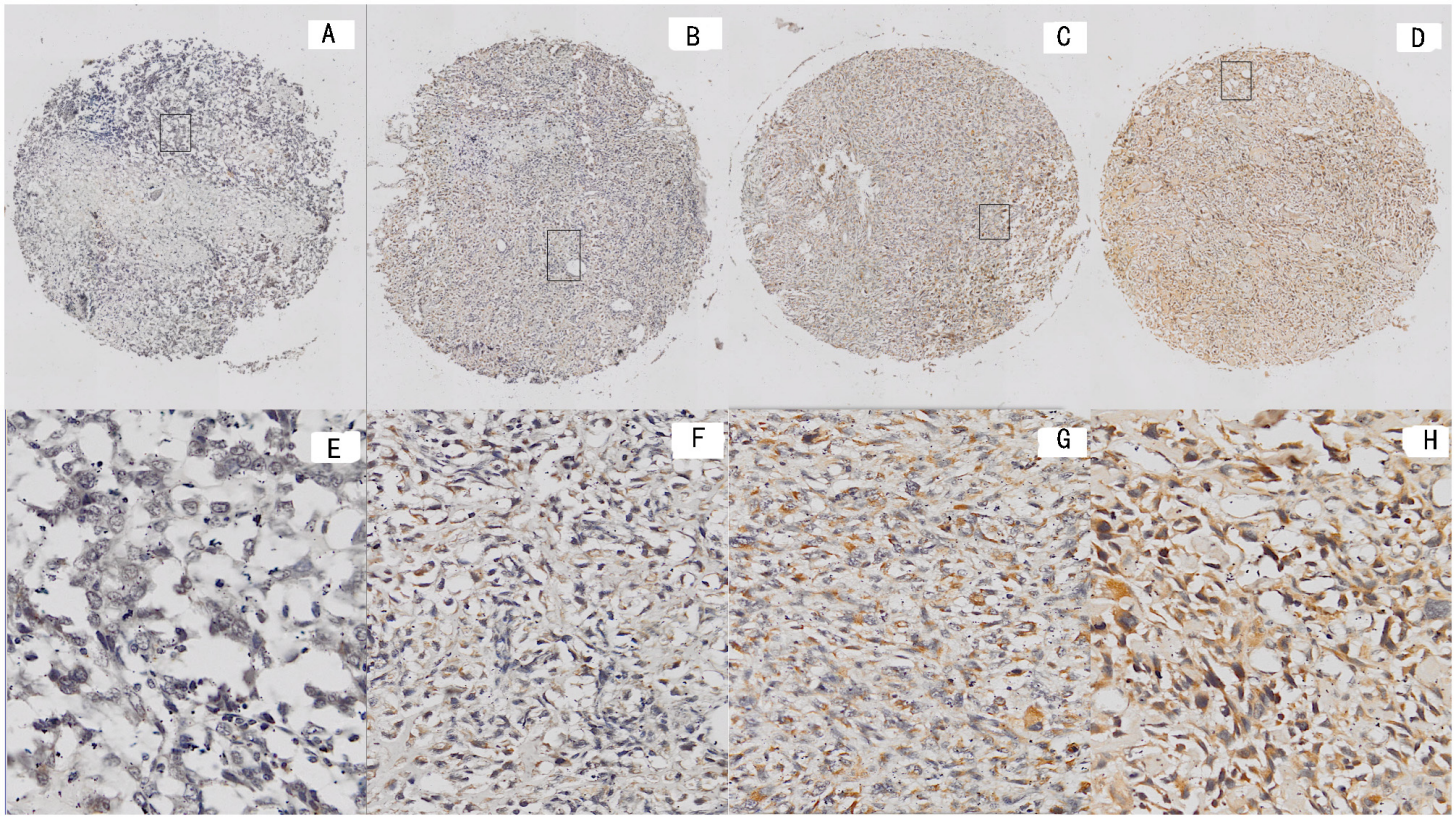
Representative immunohistochemical staining forPLA2G16 in tissue microarray. Representative PLA2G16 staining samples at magnification of 40at levels of 0, 1, 2, and 3 (A), (B), (C) and (D).RepresentativePLA2G16 staining samples at magnification of 200 at levels of 0, 1, 2, and 3 (E), (F), (G) and (H).

Positive PLA2G16 IHC staining was mainly present in the cytoplasm of the tumor cells (Fig 2A–2H).Of the 119 osteosarcoma patients, positive PLA2G16 expression was observed in 65 patients (54.6%).The PLA2G16 expression levels in the whole tumor sections were entirely consistent with the results from the TMA analysis. To elucidate the biologic significance, we investigated the association of patient clinicopathologic features and PLA2G16 expression levels. Although positive staining of PLA2G16 was present in both non-metastatic and metastatic tumors, the percentage of PLA2G16-positive staining in osteosarcoma with metastases (38/50, 76.0%) was significantly higher than in the samples without metastases (27/69, 39.1%; P<0.01, Table 1). Positive PLA2G16 expression was more frequent in osteosarcoma tissues with metastasis (P<0.01, X^2^ test). These data were consistent with the qRT-PCR analyses. However, no significant difference was observed between the expression of PLA2G16 and patient gender, age, tumor location, histological classification, histological grade and Enneking staging.

### Expression of PLA2G16 protein is associated with poor prognosis in patients with osteosarcomas

**Fig 3:**
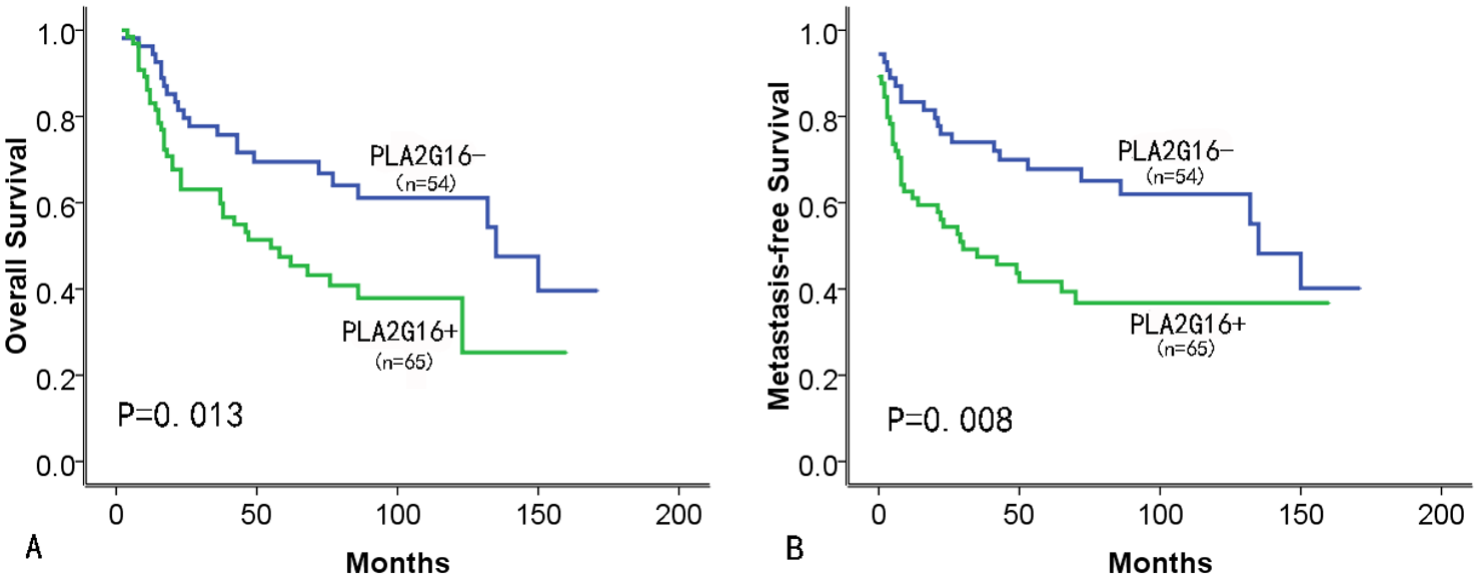
Positive PLA2G16 immunohistochemical staining was associated with poor prognosis and survival. (A) Overall survival (OS)and (B) metastasis-free survival (MFS) of PLA2G16 positive or negative patients was determined by Kaplan-Meier analysis.

Further analyses of the patient samples indicated that the 3-year OS and MFS rates were 68.8% and 59.7% for the total study population, respectively. Importantly, the negative PLA2G16 group had significantly greater survival rates for 3-year OS and MFS than did the positive PLA2G16 group (75.7% vs. 63.1%, P<0.02, Fig 3A, and 74.0% vs. 47.4%, P<0.01, Fig 3B, respectively) by the Kaplan—Meier method and a log-rank test. Interestingly, histological grade and Enneking staging were still significant prognostic factors for both OS and MFS (P<0.01; Table 2), suggesting that PLA2G16 was an independent biomarker for OS and MFS in addition to histological grade and Enneking staging. We then performed multivariate Cox regression analysis, which revealed that PLA2G16expression, histological grade and Enneking staging were indeed independent prognostic factors for OS and MFS (P<0.05; Table 3).These data indicated that PLA2G16 may be a significant and novel biomarker for evaluating the prognoses of osteosarcoma patients.

**Table 3:** Multivariate analysis of factors associated with overall survival and metastasis-free survival.

Variable	HR (95% CI)	P value
OS		
PLA2G16(+vs-)	1.857 (1.088–3.168)	0.023
Histological grade	1.711 (1.165–2.541)	0.006
Enneking staging	2.596(1.602–4.208)	<0.001
MFS		
PLA2G16(+vs-)	1.976 (1.158–3.369)	0.012
Histological grade	1.612 (1.101–2.361)	0.014
Enneking staging	3.526(1.920–6.475)	<0.001

Abbreviation: HR, Hazard ratio; CI, confidence interval; OS, overall survival; MFS, metastasis-free survival; PLA2G16, Group XVI phospholipase A_2_.

## Discussion

The main cause of mortality in osteosarcoma patients is metastasis. Patients with lung or other bone metastases have a 5-year survival rate of less than 20% [2, 3].Despite the many existing treatment strategies, the survival rates for osteosarcoma with metastasis have remained unchanged over the last few decades [15]. Therefore, the study of biomarkers for osteosarcoma metastasis is important for improving the survival of patients. Here, we reported for the first time that PLA2G16 was expressed at a higher level in human osteosarcoma patients with metastasis compared with non-metastatic patients and that the positive PLA2G16 expression in osteosarcoma tissues was significantly correlated with metastatic features and with shorter OS and MFS times. Previously, *PLA2G16* was reported to be a crucial gene for obesity development. Loss of *Pla2g16* in mice inhibited obesity in *Ob/Ob* knockout mice [16]. The link between obesity and poor cancer prognosis has recently received much attention. The underlying mechanism may be due to increased lipids and lipid signaling, inflammatory responses, insulin resistance, and adipokines [17].Epidemiological studies have shown that obesity is not only associated with an increased risk of several types of cancer, including colon, endometrial, postmenopausal breast, kidney, esophageal, pancreatic, gallbladder, liver, and hematological malignancy [18, 19] but that it also leads to poorer treatment responses and increased cancer-related mortality [20, 21]. For instance, osteosarcoma patients who have a high BMI at diagnosis have a lower overall survival compared with patients who have a normal BMI [22], which is consistent with our hypothesized role of PLA2G16 in promoting tumor progression and poor prognosis by mediating lung metastases.

Moreover, PLA2G16 generates free fatty acids, usually arachidonic acid, and lysophosphatidic acid (LPA) from phosphatidylcholine [5]. Arachidonic acid can be converted into prostaglandin E_2_ (PGE_2_)and other prostaglandins by cyclooxygenase-2 (COX2) [23], which plays a role in regulating the migratory and invasive behavior of cells during the development and progression of cancer [24].Additionally, COX-2 expression in osteosarcoma lung metastases can be used as a prognostic factor [25].Furthermore, LPA has been demonstrated to induce cell proliferation, invasion, migration and survival [7]. With the function of both free fatty acids and LPA in cancer progression and metastasis, it is not surprising to consider PLA2G16 to be an important biomarker for osteosarcoma metastasis. The possible mechanisms of PLA2G16 in osteosarcoma metastasis may be due to arachidonic acid and LPA-induced downstream signaling pathways. Additionally, LPA is known to trigger Hippo [26]and mitogenic signaling [27]. Both of these pathways contribute to tumor progression and metastasis.

Recently, we have also demonstrated that increased expression levels of PLA2G16 mediated by mutant p53 contribute to osteosarcoma progression and metastasis [4].It will be very interesting to investigate whether p53 mutations in osteosarcoma correlate with the expression of PLA2G16. Finally, this study demonstrated thatPLA2G16 can serve as an independent and significant prognostic factor of OS and MFS. Thus, PLA2G16 may be a new therapeutic target for metastatic osteosarcoma patients in the future.

## Supporting Information

S1 Table Detailed survival information of 119 osteosarcoma patients. (XLSX)
